# Molybdenum Disulphide Modified Polylactide for 3D Printed (FDM/FFF) Filaments

**DOI:** 10.3390/polym15102236

**Published:** 2023-05-09

**Authors:** Maciej Kujawa, Julia Głowacka, Wojciech Pawlak, Bogna Sztorch, Daria Pakuła, Miłosz Frydrych, Justyna Sokolska, Robert E. Przekop

**Affiliations:** 1 Faculty of Mechanical Engineering, Wroclaw University of Science and Technology, Wybrzeże Stanisława Wyspiańskiego 27, 50-370 Wroclaw, Poland; maciej.kujawa@pwr.edu.pl (M.K.); w.pawlak@pwr.edu.pl (W.P.); justyna.sokolska@pwr.edu.pl (J.S.); 2 Faculty of Chemistry, Adam Mickiewicz University in Poznań, Uniwersytetu Poznańskiego 8, 61-614 Poznan, Poland; julia.glowacka@amu.edu.pl (J.G.); darpak@amu.edu.pl (D.P.); frydrych@amu.edu.pl (M.F.); 3 Centre for Advanced Technologies, Adam Mickiewicz University, Uniwersytetu Poznańskiego 10, 61-712 Poznan, Poland

**Keywords:** polylactic acid (PLA), 3D printing, Fused Deposition Modelling (FDM), molybdenum(II) sulphide

## Abstract

MoS_2_ is an additive used to improve the tribological properties of plastics. In this work, it was decided to verify the use of MoS_2_ as a modifier of the properties of PLA filaments used in the additive FDM/FFF technique. For this purpose, MoS_2_ was introduced into the PLA matrix at concentrations of 0.025–1.0% by weight. Through extrusion, a fibre with a diameter of 1.75 mm was obtained. 3D printed samples with three different filling patterns were subjected to comprehensive thermal (TG, DSC and HDT), mechanical (impact, bending and strength tests), tribological and physicochemical characteristics. The mechanical properties were determined for two different types of fillings, and samples with the third type of filling were used for tribological tests. Tensile strength has been significantly increased for all samples with longitudinal filling with improvement up to 49%. In terms of tribological properties, higher values of the addition (0.5%) caused a significant increase of up to 457% of the wear indicator. A significant improvement in processing properties in terms of rheology was obtained (416% compared to pure PLA with the addition of 1.0%), which translated into more efficient processing, increased interlayer adhesion and mechanical strength. As a result, the quality of printed objects has been improved. Microscopic analysis was also carried out, which confirmed the good dispersion of the modifier in the polymer matrix (SEM-EDS). Microscopic techniques (MO, SEM) allowed for the characterization of the effect of the additive on changes in the printing process (improvement of interlayer remelting) and to assess impact fractures. In the tribological area, the introduced modification did not bring spectacular effects.

## 1. Introduction

Polylactide (PLA) is a biodegradable polymer that is used as an alternative to petroleum-based polymers [1,2]. It is a thermoplastic obtained by the bioconversion and polymerization from natural raw materials such as corn meal [3]. It has comparable tensile strength, stiffness and gas permeability to polymers derived from fossil fuels [4,5]. PLA is used in drug carriers, food packaging, film and 3D printing [6,7]. Three-dimensional printing is widely used in both research and commercial productions [8,9,10]. The most widely known and used 3D printing method is fused filament fabrication (FFF)/fused deposition modelling (FDM) technology. Using this technique, shapes of standardized sizes can be printed and subjected to a series of tests to characterize their physical and FVchemical properties. In this work, molybdenum(II) sulfide was used as a modifier of the PLA matrix. Transition metal sulfides, i.e., MoS_2_, are semiconductors with a two-dimensional structure, which, when introduced into the polymer matrix, improves the mechanical properties, elastic modulus, strength, creep and fatigue [11,12,13]. MoS_2_ has applications as a lubricant, due to its friction-reducing properties, and also as a catalyst [14]. In recent years, studies have been conducted on the effects of MoS_2_ in polymer composites. All studies have shown improved thermal and mechanical properties of the obtained systems [15,16]. The purpose of this work was to obtain and study MoS_2_/PLA composites and determine the effect of the modifier on the physicochemical properties of the obtained systems.

Nowadays, almost every machine and device has parts made of plastic. The popularity of plastics is due to their numerous advantages such as low cost, high impact strength, low specific weight, ease of processing and colouring, and aesthetic appearance or resistance to chemical and physical agents, among others. It is estimated that about 370 million tons of plastics were produced in 2020, and experts indicate that this quantity may increase to 1.3 billion tons in 2060. This amount is increasing all the time, due to new applications for plastics continuing to be sought as well as the replacing of components made from other materials such as metals. Plastics are beginning to be used to make seals, chemical-resistant coatings or even machine hydraulic components [17].

With the mass use of plastics, however, comes the serious problem of waste. Plastics that have entered the environment will remain in the environment for up to several hundred years before decomposing. Only a small portion of plastics is recycled; the rest is landfilled, or it lingers in the environment, harming people, animals and plants. Particularly dangerous is the so-called microplastic, which enters living organisms along with water [18].

In view of the problems described, special emphasis is being placed on obtaining “environmentally friendly” plastics. This term is very broad and includes plastics that are recyclable, made from raw materials of natural origin or biodegradable. Particularly, the latter group is highly desirable since a plastic component that is no longer needed would biodegrade, i.e., degrade under the influence of atmospheric agents or microorganisms into low-molecular-weight products that pose no threat to the environment.

Plastics are also used for sliding components. In addition to the advantages already mentioned, it should be noted that they can work with steel without lubricant. This gives an almost completely maintenance-free operation and protects the environment since it is estimated that about 40% of lubricants (52 million tons per year) end up in the environment as a result of failures and leaks. Despite this positive touch for the environment, unneeded sliding components become a nuisance waste. Plastics used for sliding components usually contain additives (mainly to reduce wear) making them even less recyclable. In addition, wear products—small fragments of material detached from the sliding element—are produced during cooperation, which end up in the environment and contaminate it.

The authors of this article focused on polylactide, which is one of the most widely used biodegradable plastics. PLA is a thermoplastic linear polyester [11]. It is biocompatible, easy to process and obtained from renewable raw materials. In addition, it is very suitable for 3D printing with FDM/FFF (a very popular, inexpensive and uncomplicated incremental technology) [19,20]. 

Recently 3D printed PLA has been noticed as a potential tribological material; therefore, the interest in this filed has emerged [21,22,23,24]. Research has also been conducted on 3D printed composites based on PLA, such as PLA/bronze composite [25], PLA/carbon black and PLA/alumina nanocomposites [26], and PLA/corn cob composite [27]. The authors of this article described their first trials of enriching a PLA matrix with MoS_2_ additions in one their previous publications, which proved higher content (above 1%) to be negative on the desired properties of lower friction coefficient and wear [28].

Neat PLA has a significant coefficient of friction and high wear when working with steel. For this reason, it is necessary to create composites based on it with appropriate additives. The additives should provide improved tribological properties as well as be environmentally neutral so that PLA does not contaminate the environment after biodegradation. In our first work, PLA + graphite composite was produced and tested (the material was patented) [20].

The very good results associated with the application of graphite to PLA prompted the authors of the publication to seek further additives. This lead the authors to MoS_2_, which has a layered structure (like graphite) and is environmentally harmless. This article presents the effect of the addition of molybdenum disulfide (MoS_2_) on the properties of PLA.

The goal of the research and creation of the compositions based on PLA and MoS_2_ addition is to ultimately design a material with improved mechanical, tribological and processing properties. On the one hand, it is important to decrease the wear, friction coefficient and tensile strength to assure long and failure-free work of even the prototype bearings; however, on the other hand, processing properties such as MFI should also be improved to ensure better lamination between the polymer layers when the 3D printing process is in progress. The design process of the said material requires the researchers to carefully investigate the influence of each type of additive used.

This paper presents MoS_2_/PLA composite materials, which were obtained by 3D printing with various infill patterns. The materials were subjected to physicochemical characterization in order to determine the effect of the additive on the properties of the polymer. A thermal analysis was conducted to determine thermal stability (TGA, HDT) and characteristic phase transition temperatures (DSC) as well as an analysis of hydrophilic–hydrophobic and rheological properties. Comparative mechanical tests (tensile strength, bending strength, impact strength) were carried out. The next step involved the determination of tribological properties. In order to determine the MoS_2_ dispersion in the matrix, SEM/EDS images and optical microscope images were taken. The obtained results allowed for the conclusion that the addition of MoS_2_, even at a low concentration, changes the properties of the polymer and facilitates its processing.

## 2. Materials and Methods

### 2.1. Samples Preparation

PLA-type Ingeo 2003D was purchased from NatureWorks (Minnetonka, MN, USA). The MoS_2_ additive (from Selkat Kraków, Poland) had a grain size of <20 µm with a purity of 98.19%. 

The polymer and the filler were homogenized using a ZAMAK MERCATOR WG 150/280 laboratory two-roll mill. A portion of 500 g PLA Ingeo™ 2003 D was mixed with MoS_2_, until the final concentration of the additive of 5.0 wt%. The mixing was performed for 15 min when the rolls’ temperature reached 210 °C until full homogeneity of the concentrates. Masterbatch was granulated by a WANNER C17.26 sv grinding mill. The granulates were diluted with neat PLA up to the final filler concentrations of 0.025, 0.05, 0.1, 0.25, 0.5 and 1.0 wt% upon extrusion moulding of a stream with consequent cold granulation on the HAAKE Rheomex OS twin-screw extrusion setup line, and then dried for 24 h at 40 °C.

The granulates obtained as above were used for moulding of filaments of 1.75 mm in diameter by a single-screw extrusion setup FILABOT EX6 FILAMENT EXTRUDER. Using a FlashForge Finder 3D printer, three types of samples were printed by FDM/FFF: oars and bars, according to PN-EN-ISO 527-2, and pin samples. Parameters of printing are given in Table 1.

### 2.2. Methods

#### 2.2.1. Mechanical Properties

For flexural and tensile strength testing, specimens were 3D printed with dimensions in accordance with the requirements of PN-EN ISO 178 and PN-EN ISO 527. The experiments were conducted according to the listed standards. Tests of the obtained specimens were performed on a INSTRON 5969 universal testing machine with a maximum load force of 50 kN. The traverse speed for tensile strength measurements was set at 2 mm/min, and the flexural strength was also set at 2 mm/min.

Charpy impact test (with no notch) was performed on a Instron Ceast 9050 impact-machine according to PN-EN ISO 179. For all the series, 7 measurements were performed.

#### 2.2.2. Rheology

The effect of the modifier addition on the mass flow rate (MFR) was also determined. The measurements were made using a Instron plastometer (Norwood, MA, USA), model Ceast MF20 according to the applicable standard of PN-EN ISO 1133. The measurement temperature was 190 ± 0.5 °C, while the piston loading was 2.16 kg.

#### 2.2.3. Contact Angle Analysis (WCA)

Contact angle analysis (WCA) was performed by the sessile drop technique at room temperature and atmospheric pressure with a Krüss DSA100 goniometer (Hamburg, Germany). Three independent measurements were performed for each sample, each with a 5 µL water drop, and the obtained results were averaged to reduce the impact of surface nonuniformity.

#### 2.2.4. Thermal Analysis

Thermogravimetry (TG) was performed using a NETZSCH 209 F1 Libra gravimetric analyzer (Selb, Germany). Samples of 5 ± 0.2 mg were cut from each granulate and placed in Al_2_O_3_ crucibles. Measurements were conducted under nitrogen (flow of 20 mL/min) in the range of 30–800 °C and a 10 °C/min heating rate. Differential scanning calorimetry (DSC) was performed using a NETZSCH 204 F1 Phoenix calorimeter. samples of 6 ± 0.2 mg were cut from each granulate and placed in an aluminium crucible with a punctured lid. The measurements were performed under nitrogen in the temperature range of 20–290 °C and a 10 °C/min heating rate.

Heat distortion temperature (HDT) tests were carried out on specimens with dimensions corresponding to the flexural beam (4 × 10 × 80 mm), and the test was carried out in accordance with PN-EN ISO 75, HDT-A (1.8 MPa). For all measurement series, three measurements each were taken, and the result averaged.

#### 2.2.5. Microstructure Characteristics

SEM microphotographs of the breakthroughs after the impact test and SEM-EDS elemental analysis of the distribution of molybdenum disulphide in the composite by Mo mapping were taken using a Quanta FEG 250 (FEI) high-resolution scanning electron microscope.

Surface structure and breakthroughs were analysed under a Digital Light Microscope Keyence VHX 7000 with 100× to 1000× VH-Z100T lens (Osaka, Japan). All of the pictures were recorded with a VHX 7020 camera.

#### 2.2.6. Tribological Tests

**Figure 1:**
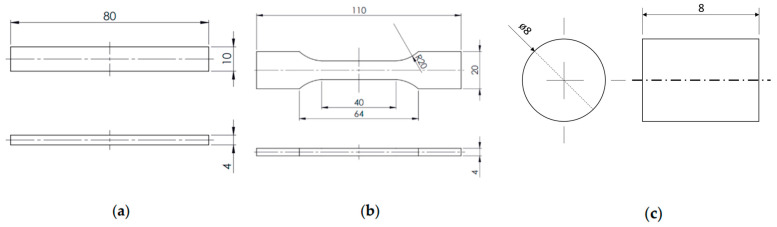
Dimensions of specimens used for mechanical ((**a**)—bending, (**b**)—tensile) and (**c**) tribological tests given in mm.

The tribological tests were performed on a pin-on-disc stand. The test parameters are presented in Table 2, and the position is shown in Figure 1. The wear rate was measured using a micrometre. The height of the sample was checked before cooperation and compared to that obtained after cooperation (dimensions after cooperation were measured only after the temperature of the sample stabilized). Four samples from each material were tested. The value of the friction force was recorded by the computer during the whole test.

## 3. Results and Discussion

### 3.1. Mechanical Properties

#### 3.1.1. Tensile Test

In order to investigate the effect of the addition of MoS_2_ on the mechanical properties of composites based on PLA, comparative strength tests were performed with different contents of modifiers as well as a type of longitudinal and hexagonal honeycomb filling. As the results show, the structure of the objects significantly affects the strength properties of the printed objects, which is consistent in the literature data [29]. It can be seen that the introduction of the MoS_2_ modifier greatly influenced the general mechanical properties of PLA-based composites. Table 3 shows the changes in tensile strength caused by the addition of different amounts of MoS_2_ in polylactide matrix.

**Figure 2:**
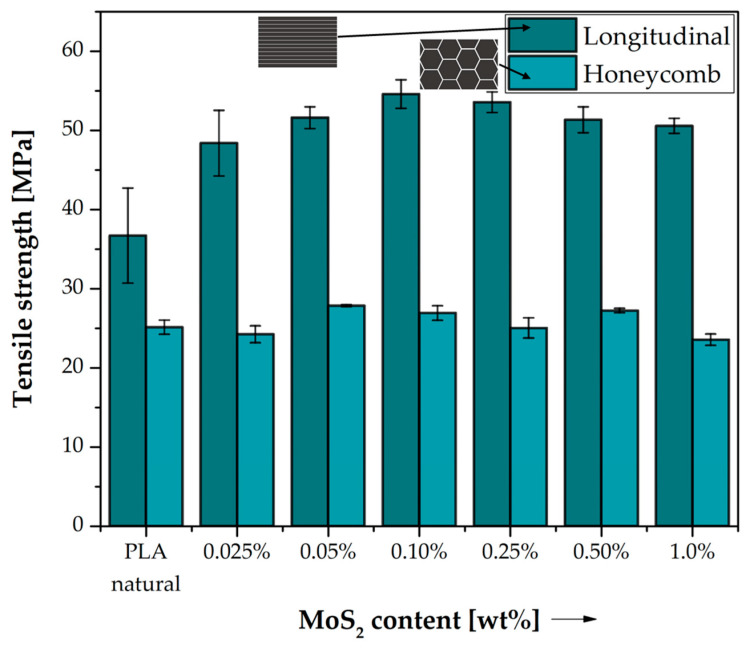
Tensile strength of neat PLA and MoS_2_/PLA composite samples. Comparison of the effects of two different 3D printed structures on their properties.

The tensile strength results are shown in Figure 2. The influence of the print structure is clearly noticeable for both neat PLA and modified PLA/MoS_2_ systems. Samples with longitudinal filling have a higher tensile strength than those with honeycomb filling in the entire concentration spectrum of the modifier used, which may result from the favourable arrangement of the paths (fibres) in accordance with the direction of the stretching axis. As shown in the study by Gonabadi, H. et al. any arrangement of tracks in the direction of the stretching axis results in unidirectional strengthening of the structure of the structure [30]. The increase in tensile strength by 11.7 MPa for this type of filling in relation to natural PLA is already clearly visible with a small mass fraction of the modifier used (0.025% by weight). The tensile strength of longitudinally filled samples increases after adding MoS_2_ to polylactide and reaches the highest value of 54.60 MPa for the addition of 0.10% MoS_2_ by weight. Then, with the increase in the addition of molybdenum sulphide, a slight decrease in strength is observed and for 1.0 wt. when the modifier is 50.60 MPa, which means that the optimal amount of the modifier for this type of composite is already reached at 0.10% modifier by weight.

The effect of the modifier addition on the strength of the samples with honeycomb filling was negligible. The highest value of tensile strength (27.85 MPa) was recorded for the content of 0.05% MoS_2_ by mass, which was an increase of only approx. 4 MPa compared to neat PLA. This effect may be caused by a small share of the material itself in the structure of the sample due to the lower degree of filling, constituting 30% of the volume of the printed object. With 100% infill, failure of the specimen occurs due to material discontinuity, while for hexagonal specimens with 30% infill, an increase in strength is not observed with a change in sulphide modifier content. As failure occurs by breaking the adhesion between the infill paths, samples with 100% infill fail due to material discontinuities, while samples with 30% infill fail due to discontinuities in the three-dimensional structure.

The honeycomb structure used in this study is constructed of regular hexagons that are repeated regularly and form a single honeycomb cell. The cells are arranged at a constant distance from each other, creating a characteristic “honeycomb” structure, and the space between the individual elements is responsible for the specific carrying or energy-absorbing properties of this structure. At the very beginning, the structure of the entire patch filling the sample is deformed. Individual cells are stretched in the direction of the force, but do not break immediately, as evidenced by the greater elongation noted for the corresponding tensile strength than in the longitudinally filled samples. The mechanism of load transfer in the case of the described types of fillings is completely different. Samples with longitudinal filling break brittlely due to the presence of many small surfaces corresponding to the cross-sectional area of single paths arranged densely next to each other, while samples with honeycomb filling are destroyed as a result of more plastic deformations, despite them being able to carry loads of smaller values, as indicated by the tensile strength results obtained in our strength tests. Generally, during uniaxial stretching, the diameter of individual fibres decreases and debonding occurs between individual fibres, which, due to limited interlayer adhesion, leads to their separation from each other. The addition of molybdenum sulphide had a positive effect on increasing the penetration between the layers, which is why samples with longitudinal filling are characterized by greater deformation than those made with pure PLA [31,32].

**Figure 3:**
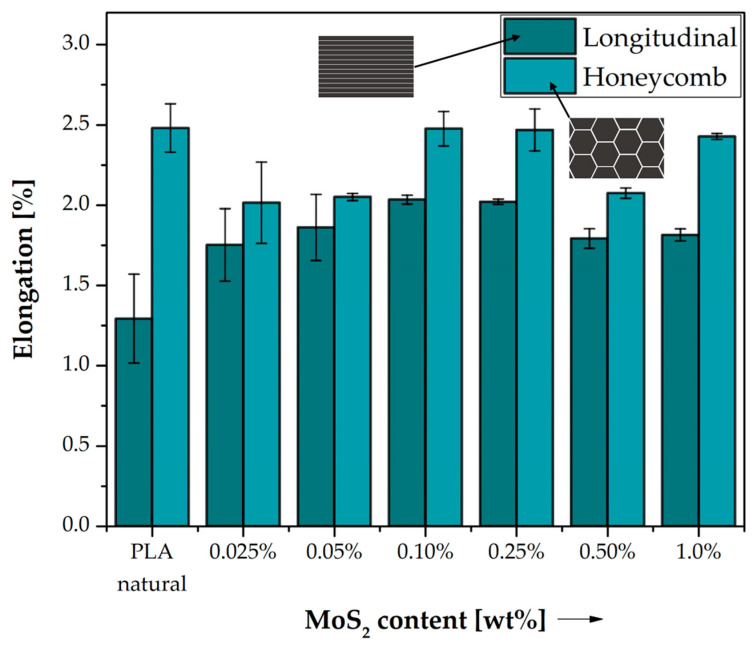
Elongation corresponding to the tensile strength of neat PLA and composite samples. Comparison of the effects of two different 3D printed structures on their properties.

Figure 3 shows the results of the static tensile test for the obtained MoS_2_/PLA composites. In the case of reference samples, significantly higher tensile strength strain values are observed for honeycomb samples made of pure PLA than those with longitudinal filling (ε = 1.29%). This difference is about 1.2%, i.e., the deformation obtained for PLA honeycomb samples is practically 100% greater than those with longitudinal filling. As noted earlier, the load transfer mechanism for the infill structures used varies. The honeycomb structure, due to its cellular structure, is a structure capable of greater plastic deformation. It is worth noting that, despite the lower percentage of material used in the test fittings, they are capable of greater deformation than fittings containing 100% longitudinal filling. In the case of modified samples, these differences are much smaller. The percentage change in the deformation of the modified samples in relation to pure PLA was calculated for each of the filling patterns and presented in Table 4. By analysing the effect of material modification on the strain of the samples under tension, it can be concluded that in the case of longitudinal filling, the strain value increases with the concentration of the modifier in PLA and for 0.10 wt% MoS_2_, the strain corresponding to Rm is about 2%, then a slight decrease is observed with increasing modifier in the material structure, and the strain for 1.0 wt% MoS_2_ in PLA is 1.8%. For honeycomb-filled samples, there is no consistent relationship between the concentration of the modifier used. Initially, the MoS_2_ fractions in PLA (0.025–0.05 wt%) show a decrease in tensile strain, while for the next two concentrations, these values increase and remain close to the pure polymer printed in a pattern with the same structure. The highest deformation value for the honeycomb structure was recorded for a concentration of 0.10 wt%. MoS_2_ (2.48%), which also corresponds to the highest tensile strength of the tested samples. The lack of a constant trend accompanying the change in the concentration of the modifier in PLA is often found in the case of additive technologies and is associated with the presence of air gaps and other defects in the structure of the samples arising in the printing process, which then affect the deterioration of the functional properties of the materials.

**Figure 4:**
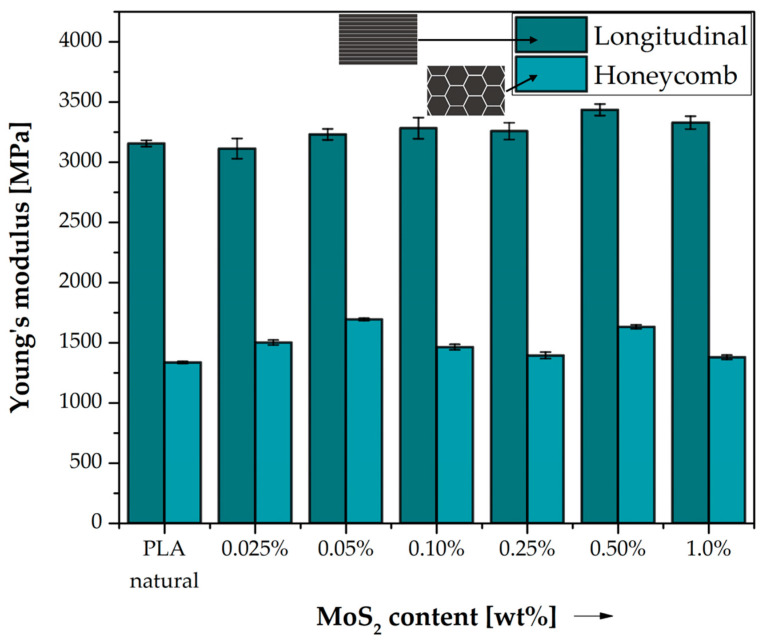
Young’s modulus of neat PLA and composite samples. Comparison of the effects of two different 3D printed structures on their stiffness.

Figure 4 compares the Young’s modulus for the tested series of samples with both types of filling in the full spectrum of MoS_2_ content in PLA. The percentage change in the Young’s modulus of the modified samples in relation to pure PLA was calculated for each of the filling patterns and presented in Table 5. Samples with longitudinal filling have much higher stiffness. The difference in stiffness between the two structures is more than twice for pure PLA and longitudinal filling E = 3155.61 MPa, and for the E honeycomb filling, it is only 1336.93 MPa. Significant differences in the stiffness of both structures result mainly from the difference in filling density. Cristian and Dudescu, in their work, examined the impact of various printing conditions on the strength parameters of objects printed in 3D using the FDM/FFF technique. In their experimental studies, they showed that Young’s modulus increases with the filling density [33]. The relatively high stiffness of the samples printed with an elongated filling also causes that such an object behaves more brittlely at break than with a honeycomb filling. The highest stiffness is observed for samples with longitudinal type filling with 0.50% MoS_2_ by weight in PLA, which is 3435.02 MPa. Molybdenum disulphide, as shown by the results of our rheological tests (Section 3.2.), has significantly increased the MFI melt flow index, which has a direct impact on the processability of polymer materials, which is directly related to achieving better print quality and properties. The increase in stiffness may be dictated by the increased cohesion of the layer that results from the modification of the polylactide. Molybdenum disulphide improves the rheological properties of PLA, which may result in better adhesion of the individual print paths to each other [34].

For the remaining concentrations of modifiers, the increase in stiffness is visible for all tested systems of this type after the introduction of molybdenum sulphide to polylactide. A slight increase in the stiffness of the tested composite systems in the full range of sulphide modifier concentrations is also observed in the case of the use of a honeycomb filling. The maximum stiffness is achieved by samples with 0.05 wt% MoS_2_ in PLA, which is 1693.75 MPa. The decrease in stiffness of the materials obtained above 0.25 wt% of the modifier in the matrix coincides with an increase in the corresponding strain relative to the reference material, which may indicate the plasticizing effect of the additive used. A general trend of a decrease in strength properties such as modulus and tensile strength above this MoS_2_ content was observed. Aggravation of the mechanical properties may result from molybdenum disulfide grouping into agglomerates, which reduces the contact surface of the modifier with the polymer matrix, and thus, the ability of molybdenum disulfide to reinforce polylactide deteriorates [35].

#### 3.1.2. Flexural Test

**Figure 5:**
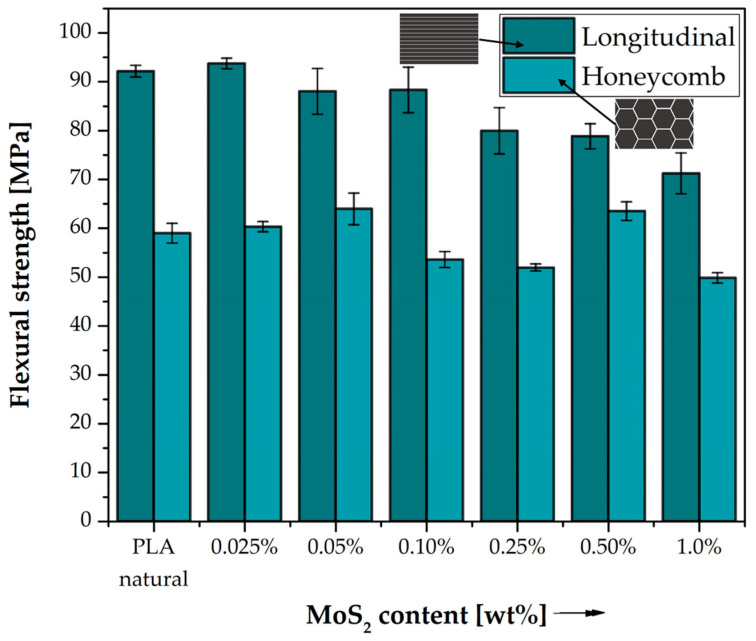
Flexural strength of neat PLA and composite samples. Comparison of the effects of two different 3D printed structures on mechanical strength obtained from flexural experiment.

Table 6 and Table 7 present the percentage differences between the results obtained in the three-point bending test for the modified specimens in relation to PLA. Figure 5 shows the dependence of flexural strength as a function of the change in MoS_2_ content in the polylactide. Comparing the results of flexural strength for neat PLA printed in the two structures discussed above, the longitudinal-filled samples have a significantly higher flexural strength (92.18 MPa) than the honeycomb type (58.99 MPa). Saniman, Muhammad Nur Farhan et al., in their work, examined the effect of different types of infills, including linear and honeycomb, on the flexural properties of components printed from polylactide using FDM/FFF technology, and also observed lower flexural strength values for honeycomb-filled samples compared to linear-filled samples [36]. For longitudinal infill at 0.025 wt% MoS_2_ content in PLA, a slight increase in flexural strength to 93.77 MPa is seen, and then a decrease in flexural strength is observed with increasing molybdenum sulfide content in PLA, which reaches its lowest value at 1.0 wt% (71.26 MPa). In the case of the honeycomb fill for MoS_2_ concentrations of 0.025 wt%, 0.05 wt% and 0.50 wt%, a slight increase in flexural strength is seen and takes the highest value of 63.98 MPa for 0.05 wt% MoS_2_. The effect of the filling used is evident throughout the cross-section of the tested materials. The flexural strength is lower for honeycomb-filled specimens than for longitudinal-filled specimens for all concentrations of molybdenum disulfide in the polylactide. Thus, it can be concluded that the structural factor significantly influences the properties of the objects produced. The arrangement of the layers and its type as well as the actual contribution of the material to the overall object determines its bending properties.

**Figure 6:**
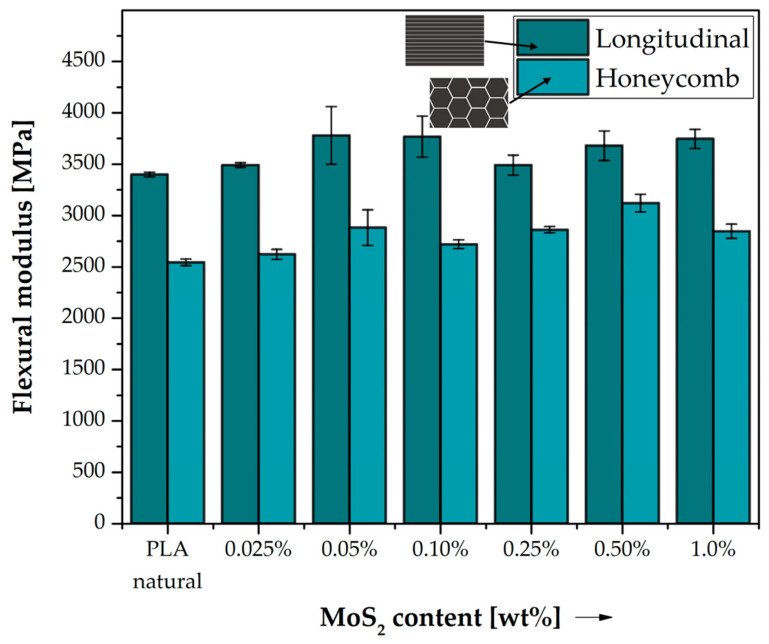
Flexural modulus of neat PLA and composite samples. Comparison of the effects of two different 3D printed structures on elasticity modulus obtained from flexural experiment.

The flexural stiffness of the tested samples is shown in Figure 6. Based on the results of the tests, a favourable effect of molybdenum sulfide on the bending stiffness of the printed samples relative to the reference material is evident. The reason for this behaviour of the material under bending forces may be due to the lamellar structure of the modifier, which hinders the free movement of the polymer chains; however, the use of honeycomb filling has a significant effect on the reduction of bending stiffness for all tested systems relative to samples with longitudinal filling. The difference in stiffness of specimens with different types of filling is largely dictated by the degree to which the material fills the specimen, which is 100% for longitudinal type specimens and 30% for honeycomb [37]. Similarly, as in the case of flexural strength, the flexural modulus increases for the longitudinal type specimens after introducing a small amount of MoS_2_ into PLA, and for 0.025 wt%, it reaches a value of 3491.80 MPa to then continue the downward trend and reach the lowest value of flexural modulus for 1.0 wt% MoS_2_ in the polylactide with a value of 3747.18 MPa. 

The flexural stiffness of honeycomb-filled specimens increases after modification with molybdenum sulfide. The highest modulus of stiffness for this type of fill was recorded for a content of 0.50 wt% MoS_2_ (3121.28 MPa).

#### 3.1.3. Impact Strength Results

**Figure 7:**
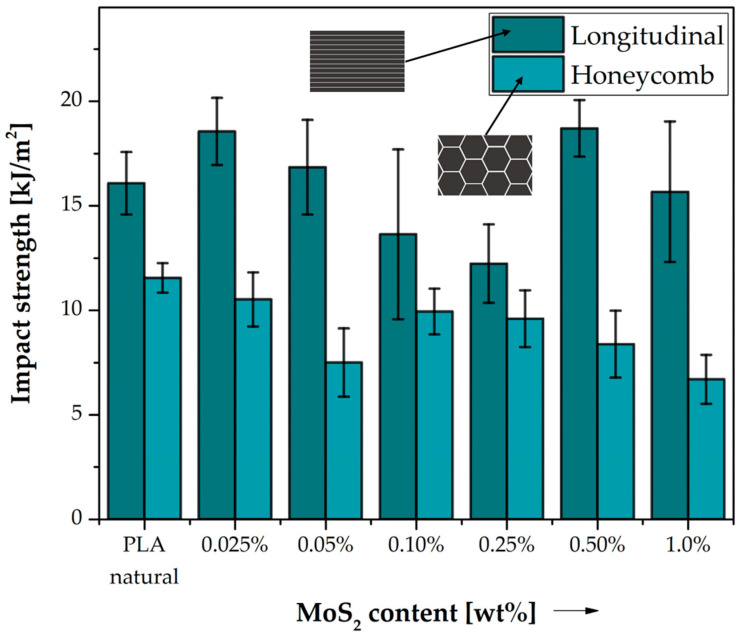
Impact strength of neat PLA and composite samples. Comparison of the effects of two different 3D printed structures on their impact properties.

The results of the impact tests are shown in Figure 7. The introduction of molybdenum sulphide to polylactide in all concentrations for the honeycomb structure decreases the impact strength parameters in relation to pure polylactide. The lowest impact strength has samples containing 1.0 wt% MoS_2_ (6.70 kJ/m^2^). When using the longitudinal fill, a slight increase in the parameter is observed, the largest by 2.62 kJ/m^2^ for the sample containing 0.50% MoS_2_ by weight. The results of the impact tests of printed samples made of pure polylactide also show that the elongated structure with a higher degree of filling generally has better impact properties compared to the honeycomb structure. The study by Subeshan, Balakrishnan et al. on the effect of filling density confirms our observations of a decrease in impact strength with a decrease in the filling density of the tested objects [38]. The differences in the impact strength of the tested samples in relation to the reference samples are presented in Table 8.

### 3.2. Rheology

**Figure 8:**
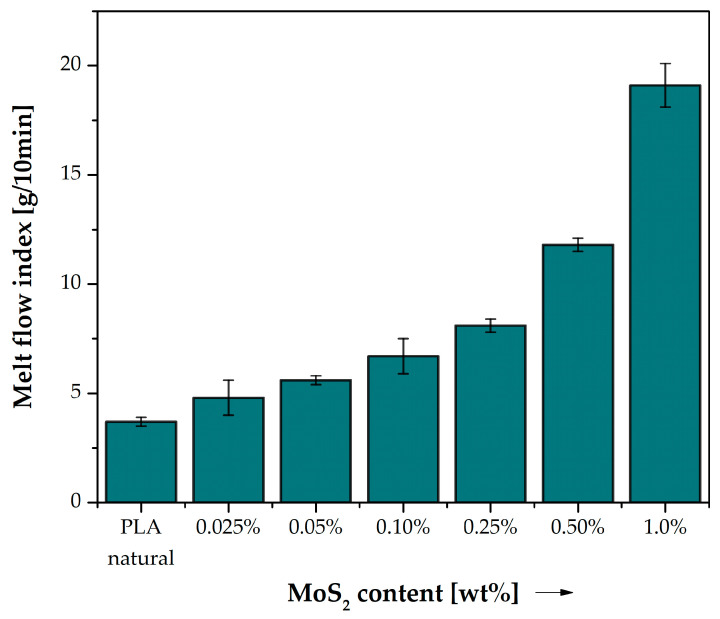
Melt flow index (MFI) of neat PLA and composite samples. Comparison of the effect of MoS_2_ content on the processing behaviour.

Table 9 shows the change in mass flow rate for different modifier contents in composites with PLA matrix compared to neat PLA. The MFR test showed that neat PLA has a relatively low MFR (about 3.7 g/10 min), which is consistent with literature reports [18]. With an increase in the MoS_2_ content in the composite structure, the MFR value increases. Figure 8 shows that even a small addition of MoS_2_ increases the flow rate of the polymer during the test. For low content of the modifier in PLA (0.025 and 0.05% by weight), the change in the MFR index is insignificant (~1 and ~2 g/10 min, respectively). The difference between MFR for pure PLA and composite with 1.0 wt% MoS_2_ at 190 °C was as much as ~15 g/10 min. Molybdenum disulfide is known for its lubricating properties, which is why it is often used as an additive to greases and oils and has also been used as a compound that improves the rheological properties of polymer blends [39]. Its lubricating properties result from the layered crystal structure. Weak van der Waals interactions between its layers and lubrication is affected by intergrain sliding [40,41]. The MFR value is an indicator of the behaviour of the material as it flows in flow channels. Such a large change in the flow rate of the melt with a negligible content of the applied sulphide modifier can have a positive effect on improving the melting of layers during the 3D printing process and, thus, also contribute to the tightness of the printed objects, improving their durability. With the increase in the content of MoS_2_ in the composite, the MFR index changes significantly. Only 0.025 wt% addition of MoS_2_ to PLA increases the MFR by 30%. Up to a content of 0.25% MoS_2_ by weight, the increase in the MFR value is almost linear. Further increasing the concentration of MoS_2_ increases the MFR almost exponentially. With a content of 0.5 wt% and 1 wt% MoS_2_, the MFR ratio is twice and four times higher than that of unmodified PLA, respectively. The addition of MoS_2_ is beneficial from the point of view of material processing, as it significantly increases the MFR index. Thanks to this, extrusion of the material requires less force and energy, which is beneficial both when processing the material with classic methods such as extrusion or injection, as well as modern methods such as 3D FDM/FFF printing. 

### 3.3. Thermal Analysis Results

**Figure 9:**
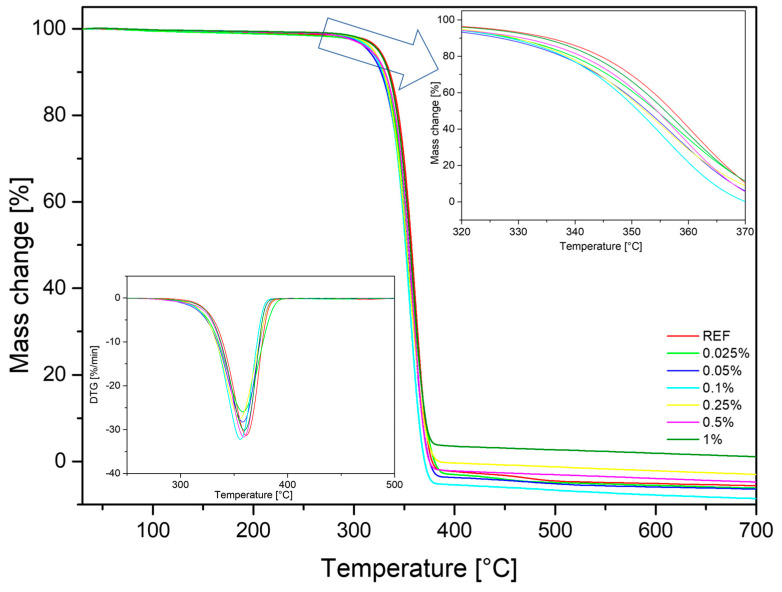
TGA and DTG curves under nitrogen atmosphere.

Thermogravimetric analysis (TGA) was carried out in air and in an inert gas to determine the influence of MoS_2_ on the thermal stability of PLA composites. The temperature of 1% mass loss, the beginning of decomposition and the temperature at the maximum rate of mass loss were determined. All data are summarized in Table 10. The TGA and DTG curves recorded for measurements in a nitrogen atmosphere (Figure 9) show the TGA curves for MoS_2_/PLS composites with different concentration of modifier in comparison to neat PLA. From both the TGA curves and the data presented in Table 10, it can be seen that the thermal decomposition of the polymer matrix with the addition of the modifier proceeded faster than in the case of neat PLA. The addition of MoS_2_ to the composites caused the diffusion of heat and gases to be disturbed. This effect does not depend on the concentration or dispersion of the modifier, the values of the beginning of decomposition and temperature at the maximum rate of mass loss are similar for all systems, and a slight increase can be observed for polymer mixtures with the highest content of additives. Given the barrier effect that limited access to heat, this caused the combustion process to end at a lower temperature.

**Figure 10:**
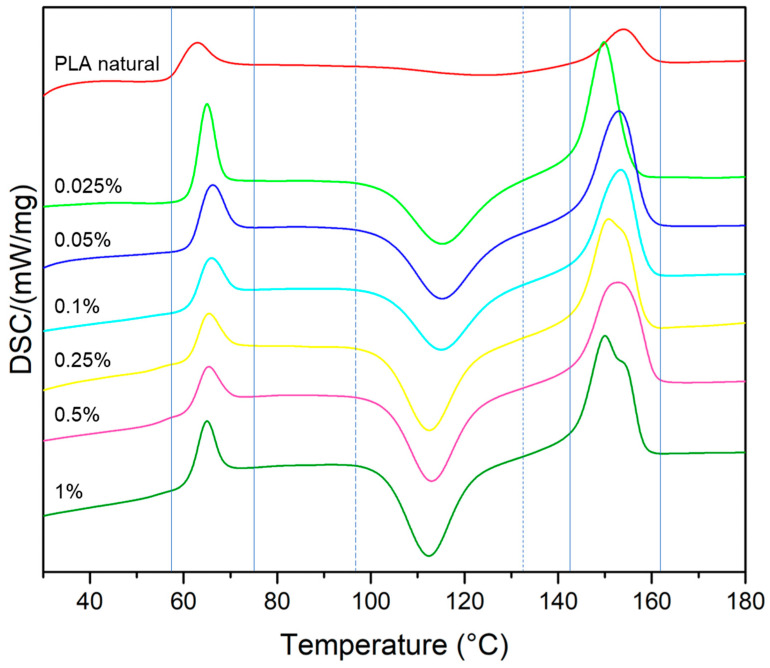
DSC curves of MoS_2_/PLA composite.

PLA and PLA/MoS_2_ composites were subjected to differential scanning calorimetry (DSC). Three characteristic phase transition temperatures for polylactide from the second heating cycle were determined (Figure 10). Each DSC curve shows the glass transition temperature T_g_ (59–70 °C), cold crystallization temperature T_cc_ (95–130 °C) and melting peak T_m_ (145–165 °C). The temperatures of the glass transition, cold crystallization and melting temperature are listed in Table 11. PLA belongs to the semi-crystalline polymers, which are characterized by an amorphous and crystalline fraction. Based on the DSC analysis, it can be seen that the addition of MoS_2_ significantly changed the character of curves. A significant effect of the additive was observed for cold crystallization. The addition of molybdenum disulfide lowered the crystallization temperature, which can be explained by the nucleation effect of the modifier. The DSC curves of PLA/MoS_2_ are characterized by a sharper T_cc_ peak compared to those of neat PLA. The glass transition temperature is higher for composite materials with the addition of molybdenum disulfide, which proves the increase in stiffness of PLA. MoS_2_ did not significantly affect the melting temperature of the polymer. 

### 3.4. Contact Angle

**Figure 11:**
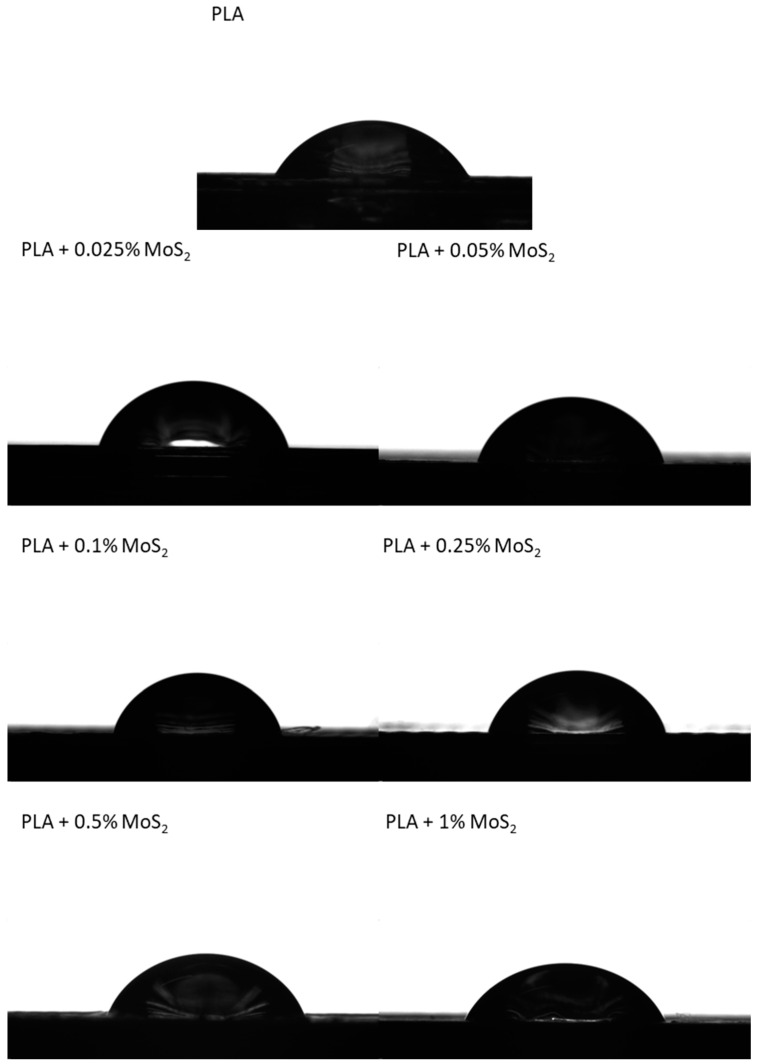
Images of water droplets during sessile drop analysis.

The contact angle analysis was carried out to determine the effect of the MoS_2_ addition on the hydrophobic properties of the composites (Table 12). The value of the contact angle of the reference sample is 63.7°. The addition of MoS_2_ caused a significant change in the values of contact angle for composites. Adding small amounts of molybdenum disulfide allowed for the increase in the value of the contact angle by about 10%. According to the literature data, MoS_2_ is characterized by hydrophobic properties [42], which results in an increase in the value of the contact angle for the modified samples compared to the PLA reference sample. The presented test results indicate high standard deviations, which is caused by the surface roughness of the materials. Figure 11 presents images of water droplets during sessile drop analysis.

### 3.5. Heat Deflection Temperature (HDT) Test

The results of the HDT analysis performed are shown in Table 13. The table compares the effect of the type of infill used on the temperature of deflection under load. The HDT values for longitudinal fill are in the range of 54.5–58.77 °C, while those for honeycomb fill are 55.76–56.54 °C. These values are therefore similar for both types of infill. It is worth noting that the use of a honeycomb structure with an infill density of only 30%, did not cause significant changes in the thermal behaviour of the samples. The modification of the polylactide with molybdenum sulfide carried out causes HDT temperature fluctuations in the range of several degrees Celsius. HDT is a neat material feature. The type of filling used and the amount of MoS_2_ introduced have virtually no effect on the thermal stability of PLA and its composites, which is largely dependent on the thermoplastic matrix. Therefore, despite noticing small differences in HDT results between the used fill structures and different MoS_2_ contents, the influence of the present factors on the temperature of deflection under load can be considered insignificant.

### 3.6. Microscopy 

#### 3.6.1. SEM-EDS

**Figure 12:**
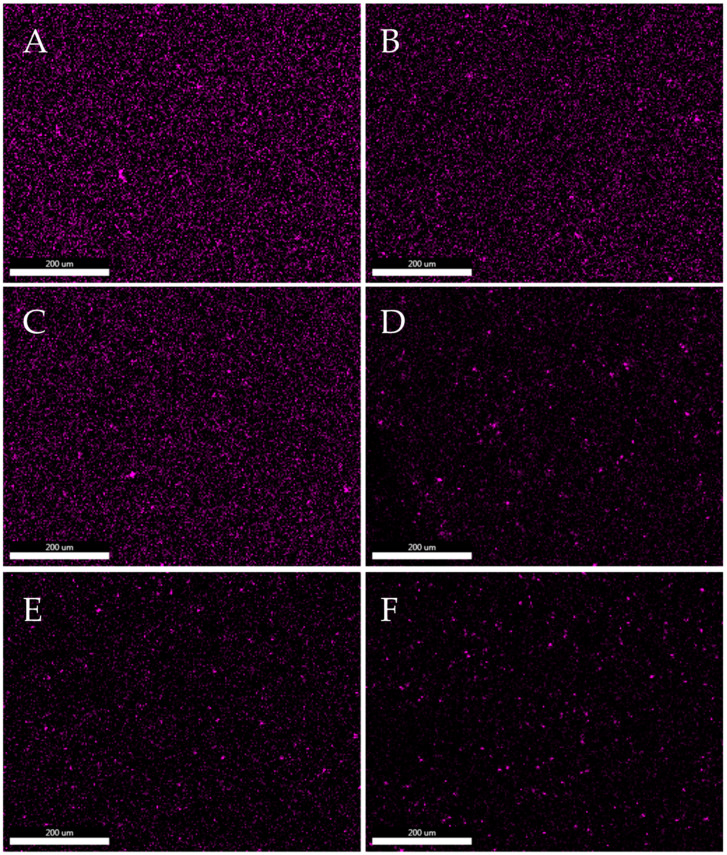
Dispersion analysis of molybdenum disulphide in a polylactide matrix. Mapping of Mo distribution in the composite structure of filament pellets with different MoS_2_ contents by SEM-EDS. ((**A**)—PLA/1.0% MoS_2_, (**B**)—PLA/0.5% MoS_2_, (**C**)—PLA/0.25% MoS_2_, (**D**)—PLA/0.10% MoS_2_, (**E**)—PLA/0.05% MoS_2_, (**F**)—PLA/0.025% MoS_2_).

**Figure 13:**
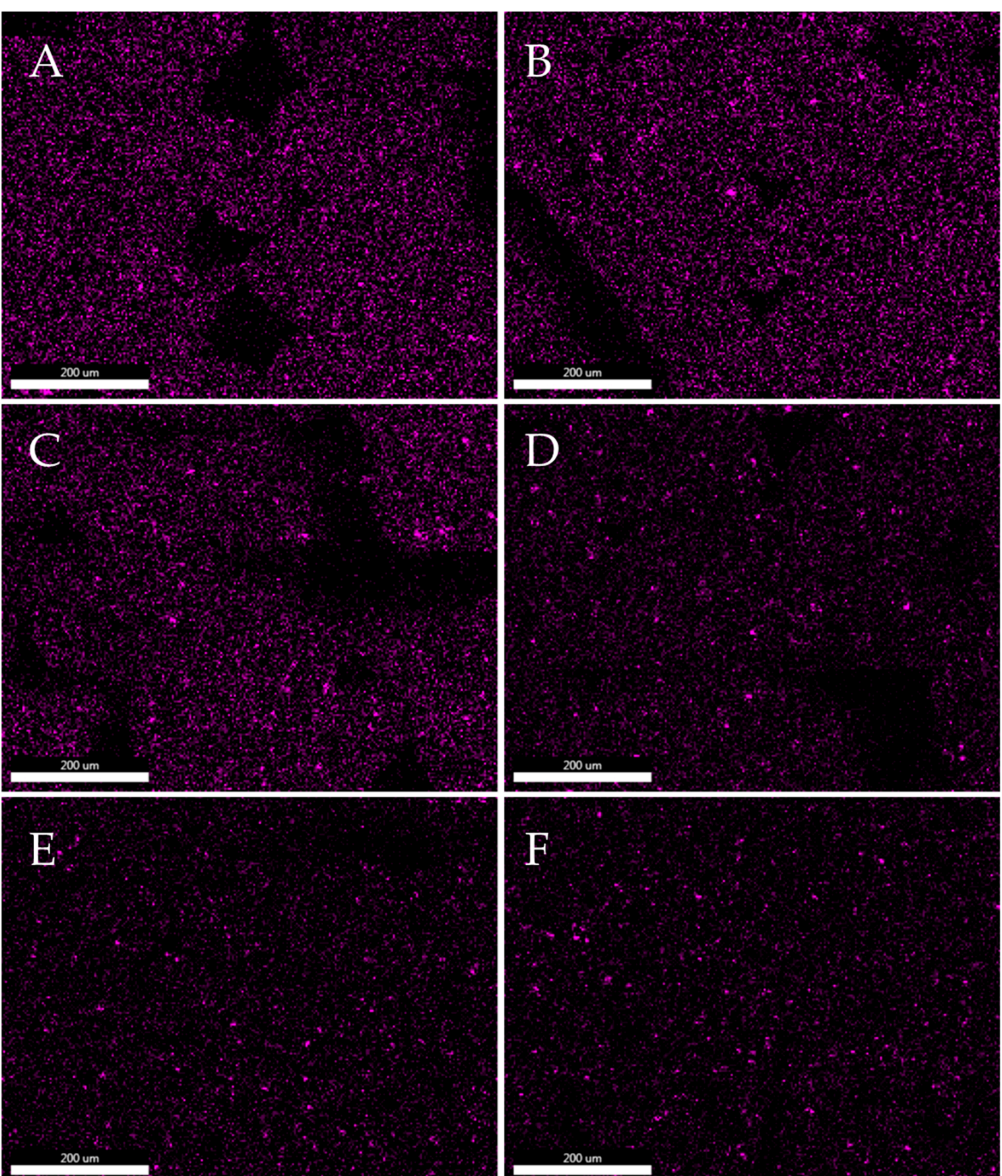
Analyses of molybdenum disulphide dispersion in a polylactide matrix. Mapping of Mo distribution in a composite structure with different MoS_2_ content using SEM-EDS. ((**A**)—PLA/1.0% MoS_2_, (**B**)—PLA/0.5% MoS_2_, (**C**)—PLA/0.25% MoS_2_, (**D**)—PLA/0.10% MoS_2_, (**E**)—PLA/0.05% MoS_2_, (**F**)—PLA/0.025% MoS_2_).

Figure 12 and Figure 13 show maps of molybdenum distribution in polylactide based on EDS elementary analysis. Molybdenum mapping determined the distribution of the modifier in the polymer matrix. The mapping was performed on the filament pellets and on the fractures after the impact test. Figure 12 and Figure 13 show that the Mo signals are evenly distributed over the entire surface. On the basis of elemental analysis, it can be concluded that the distribution of molybdenum sulphide in composites is homogenous, and the modifier is characterized by good dispersion in the polymer matrix. Similar results of molybdenum sulphide in polymer composites was noted for different matrices, e.g., polyether–ether–ketone (PEEK) [43], hydroxypropyl methylcellulose (HPMC) [44] or polyimide (PI) [45]. The high dispersion of MoS_2_ in the structure of the composites is due to the layered structure of molybdenum disulfide, thanks to which polymer chains can penetrate between the layers of the modifier. This is a feature specific to modifiers of polymeric materials with an analogous lamellar structure, such as layered silicates, e.g., MMT [46]. Both on the maps of Mo distribution made for granules and fractures, small groups of modifier molecules can be seen, however, the increase in the MoS_2_ content in the composite structure does not cause significant agglomerations of its particles. The conducted analyses also show that during the printing process (subsequent processing), there is no reagglomeration of the modifier in the polymer matrix.

#### 3.6.2. SEM

**Figure 14:**
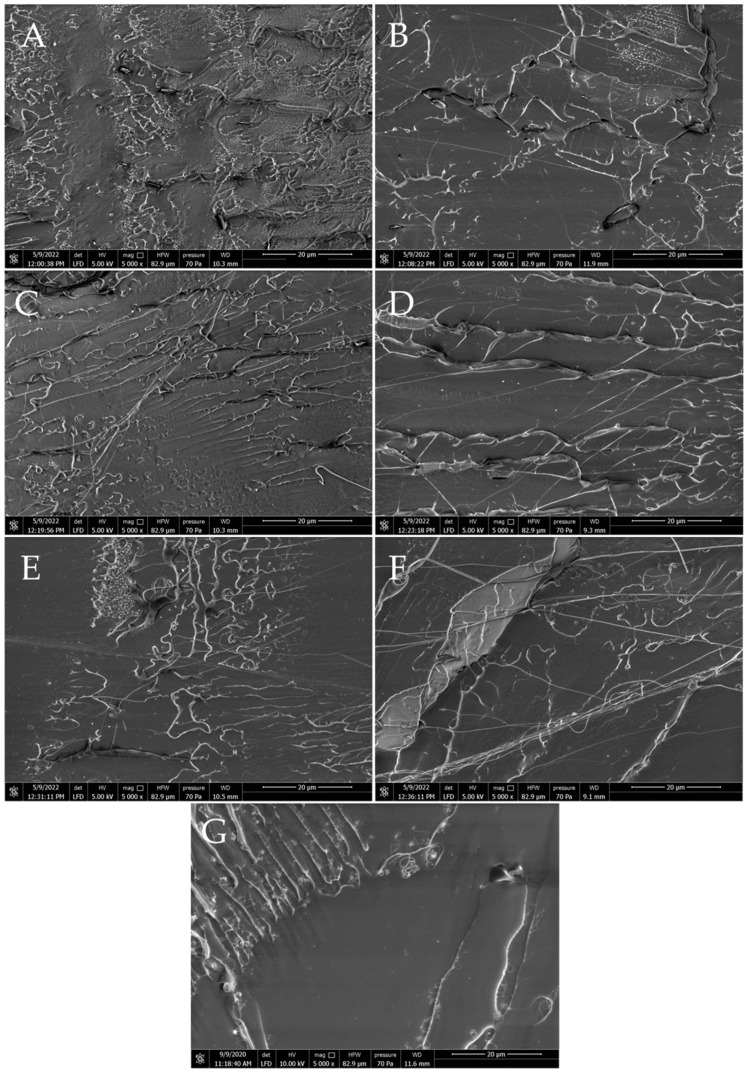
Images of PLA composite fractures after impact testing containing different wt% of molybdenum sulphide. ((**A**)—1.0% MoS_2_, (**B**)—0.5% MoS_2_, (**C**)—0.25% MoS_2_, (**D**)—0.10% MoS_2_, (**E**)—0.05% MoS_2_, (**F**)—0.025% MoS_2_, (**G**)—PLA).

SEM images were taken to characterize the morphology of the fractures and to evaluate the interfacial interactions between the modifier and the PLA matrix. Figure 14 shows a comparison of the fracture structure for PLA/MoS_2_ composites containing different MoS_2_ wt% contents. The damaged surface has smooth, wavy edges that reflect the brittle nature of PLA at room temperature. With the increase in the share of MoS_2_ in the composite structure, the texture becomes more visible in the photos, resulting from the penetration of molybdenum disulfide with the polymer material, creating some kind of path (pattern). As the filling of the composite increases, the surface of the fractures becomes rougher, which is most noticeable at the highest content of disulfide in the composite. At the same time, the existence of agglomerated MoS_2_ particles in the structure of impact fractures was not observed. The observation of the fractures allows for the conclusion that the molybdenum disulfide particles are homogeneously distributed in the polymer matrix, which is consistent with the results of the SEM-EDS analysis.

#### 3.6.3. Optical Microscopy

**Figure 15:**
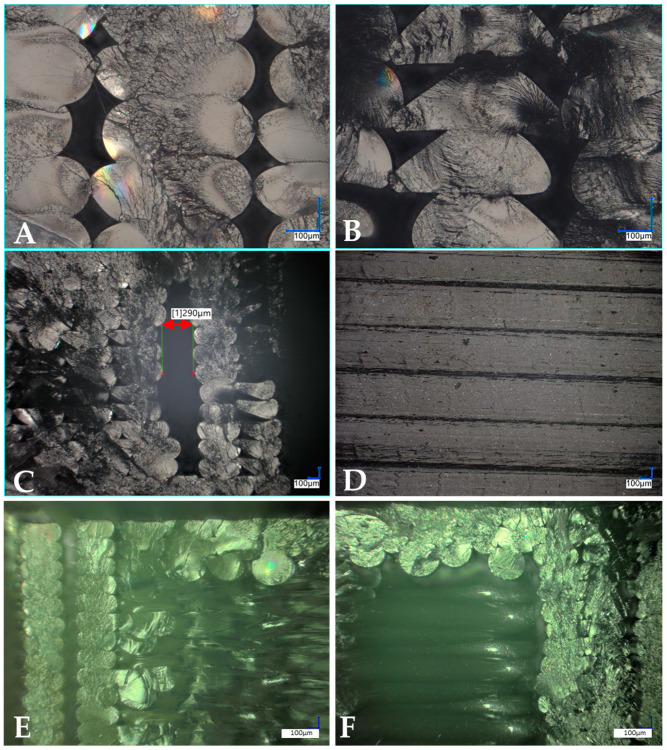
Microscopic photos of fractures and surfaces of printed fittings. Pictures (**A**–**D**) printed with longitudinal filling, (**E**,**F**) printed with honeycomb filling. (**A**)—shape and spaces between the fracture layers of the reference sample; (**B**)—shape and spaces between the fracture layers of the sample modified with MoS_2_ (0.25%); (**C**)—defect of the printed fitting; (**D**)—fitting surface; (**E**)—photo of the fracture of the reference sample; (**F**)—photo of a fracture of a sample modified with MoS_2_ (0.5%).

Samples printed with longitudinal infill have defects that are visible in the photos of sample fractures (Figure 15A). Regardless of the concentration of the additive, there are clear gaps between the applied layers of the sample. In addition, in each observed shape, an empty area of 0.2 mm to 0.4 mm can be seen, which did not have a filament layer (Figure 15C). The addition of MoS_2_ influenced the faster flow of the material. From a concentration of 0.05%, a change in the shape of the layers and greater cohesion of the material (reduction of interlayer spaces) can be seen. The surface of the tested material has a uniform structure, which indicates good homogenization of the modifier in the sample (Figure 15D). Similar results can be found for fittings printed with the honeycomb filling (Figure 15E,F).

### 3.7. Tribological Tests

**Figure 16:**
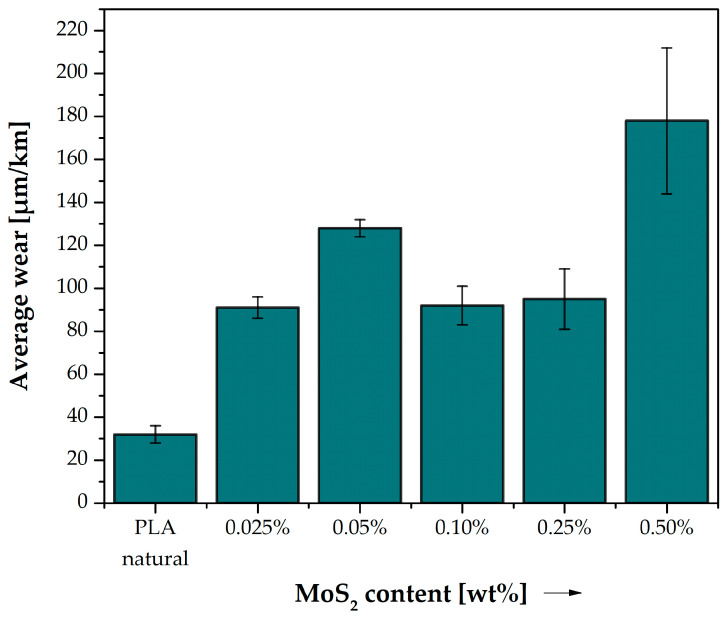
Average wear of PLA and composite samples.

Even a small addition of MoS_2_ to PLA causes a significant increase in consumption (Figure 16, Table 14). Even with a 0.025% MoS_2_ addition, the consumption increased almost twice (relative to the consumption of unmodified PLA). For a 0.05% addition, the consumption is three times higher; for 0.1% and 0.25%, it is twice as high; and for 0.5%, it is as much as 4.5 times higher. The result for the 0.05% addition can deviate from the trend due to a changing nature of the wear mechanisms, where one of the types is reaching its maximum influence with 0.05% addition, yielding, then, to different mechanism types in higher percentages. To determine the exact wear mechanism, further research needs to be conducted.

**Figure 17:**
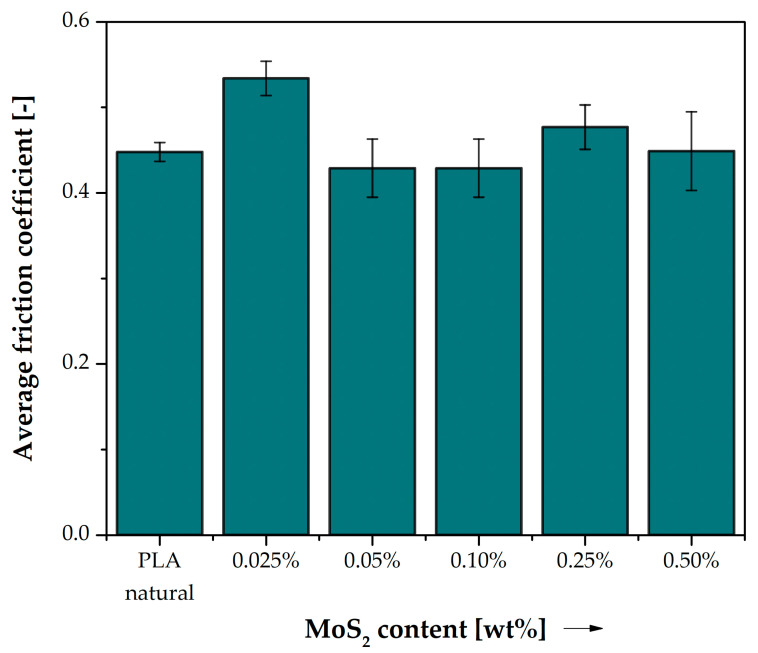
Average friction coefficient of PLA and composite samples.

In the case of the friction coefficient, only the addition of 0.025% MoS_2_ caused a significant change in the value (an increase of 19%) (Figure 17, Table 15). At higher concentrations of MoS_2_ in the composite, the value of the friction coefficient was very similar to that obtained for unmodified PLA (changes in values were up to a few percent).

## 4. Conclusions

Based on the analyses carried out, it can be concluded that MoS_2_ is an additive that significantly increased PLA consumption when working with steel but improved other properties of the samples obtained in the 3D printing process, such as tensile strength, elongation at break and Young’s modulus. In addition, MoS_2_ is also advantageous in terms of material processing as it significantly increases the castability of the material. During bending tests, MoS_2_ samples obtained lower strength than unmodified PLA. During bending, shearing occurs between the layers of the material. MoS_2_, due to its layered structure, has low shear strength (in MoS_2_, only weak interatomic interactions occur between the layers, not strong chemical bonds). It is possible that the addition of MoS_2_ weakens the material under shear. The addition of MoS_2_ changed the nature of the DSC curves, which means that it affects the phase transformations in the material and, in particular, the cold crystallization. MoS_2_ is a frequently used additive that improves the tribological properties of plastics, but in the case of a PLA-based composite, it did not work out favourably. The addition of MoS_2_ to PLA significantly worsened the abrasion resistance when working with steel. The stick-slip phenomenon was observed during tribological tests. Static and kinetic friction appeared alternately between the sample and the disc. As a result, the cooperation proceeded with high vibrations and frictional resistance. When plastic and metal work together, the plastic is transferred to the metal, creating a thin layer. As a result, there is cooperation between the plastic sample and the layer applied to the disc. As research has shown, the addition of MoS_2_ increased the strength of PLA. Since the addition of MoS_2_ increases the cohesive strength of PLA, it is very possible that the addition of MoS_2_ also increases the force between the layer applied to the metal and the sample, and this causes an increase in frictional resistance and the stick-slip phenomenon. Further research will be continued in the area of improving the lubricating properties of systems based on molybdenum disulphide and graphite. The obtained results prompt us to undertake research on other polymer matrices, such as ABS, PET or polyamide.

## Figures and Tables

**Table 1:** Process parameters that were requested when printing samples.

	Shape of a Sample and a Bar Type A	Shape of a Sample and a Bar Type B	Pin
Nozzle diameter	0.4 mm	0.4 mm	0.3 mm
Printing temperature	200 °C	200 °C	200 °C
Bed temperature	60 °C	60 °C	60 °C
Layer height	0.18 mm	0.18 mm	0.10 mm
Number of full top/bottom layers	3/3	3/3	16/16
Number of contours	2	2	3
Top and bottom layer style	Rectilinear (parallel to the long edge)	Rectilinear (parallel to the long edge)	Concentric
Infill style	Honeycomb	Longitudinal	Rectilinear
Infill percentage	30%	100%	16%
Cooling	100%	100%	100%
Printing speed	40 mm/s	40 mm/s	30 mm/s

**Table 2:** Parameters characterizing the conducted friction tests.

Parameter	Value
Pin force on the disc	5.49 N
Pin pressure on the disc—p	0.11 MPa
Disc material	Steel C45
Disc roughness	Ra = 0.35–0.45 µm
Duration of test	2 h 27 min
Linear speed in association—v	0.34 m/s
Ambient temperature—T_0_	23 °C
Friction path	3 km
Type of friction	Technically dry

**Table 3:** Tensile strength (Rm) of PLA and PLA/MoS_2_ composites.

	Neat PLA	PLA + 0.025% MoS_2_	PLA + 0.05% MoS_2_	PLA + 0.10% MoS_2_	PLA + 0.25% MoS_2_	PLA + 0.50% MoS_2_	PLA + 1.0% MoS_2_
Longitudinal infill pattern							
ΔRm	-	32%	41%	49%	46%	40%	38%
Honeycomb infill pattern							
ΔRm	-	−3%	11%	7%	0%	8%	−6%

**Table 4:** Elongation (ɛ) of neat PLA and PLA/MoS_2_ composites.

	Neat PLA	PLA + 0.025% MoS_2_	PLA + 0.05% MoS_2_	PLA + 0.10% MoS_2_	PLA + 0.25% MoS_2_	PLA + 0.50% MoS_2_	PLA + 1.0% MoS_2_
Longitudinal infill pattern							
Δɛ	-	36%	44%	58%	57%	39%	41%
Honeycomb infill pattern							
Δɛ	-	−18%	−17%	0%	0%	−16%	−2%

**Table 5:** Young’s modulus (E) of PLA and PLA/MoS_2_ composites.

	Neat PLA	PLA + 0.025% MoS_2_	PLA + 0.05% MoS_2_	PLA + 0.10% MoS_2_	PLA + 0.25% MoS_2_	PLA + 0.50% MoS_2_	PLA + 1.0% MoS_2_
Longitudinal infill pattern							
ΔE	-	−1%	2%	4%	3%	9%	5%
Honeycomb infill pattern							
ΔE	-	12%	27%	9%	4%	22%	3%

**Table 6:** Flexural strength (Rf) of PLA and PLA/MoS_2_ composites.

	Neat PLA	PLA + 0.025% MoS_2_	PLA + 0.05% MoS_2_	PLA + 0.10% MoS_2_	PLA + 0.25% MoS_2_	PLA + 0.50% MoS_2_	PLA + 1.0% MoS_2_
Longitudinal infill pattern							
ΔRf	-	2%	−4%	−4%	−13%	−14%	−23%
Honeycomb infill pattern							
ΔRf	-	2%	8%	−9%	−12%	8%	−16%

**Table 7:** Flexural modulus (Ef) of PLA and PLA/MoS_2_ composites.

	Neat PLA	PLA + 0.025% MoS_2_	PLA + 0.05% MoS_2_	PLA + 0.10% MoS_2_	PLA + 0.25% MoS_2_	PLA + 0.50% MoS_2_	PLA + 1.0% MoS_2_
Longitudinal infill pattern							
ΔEf	-	3%	11%	11%	3%	8%	10%
Honeycomb infill pattern							
ΔEf	-	3%	13%	7%	12%	23%	12%

**Table 8:** Impact strength (Re) of PLA and PLA/MoS_2_ composites.

	Neat PLA	PLA + 0.025% MoS_2_	PLA + 0.05% MoS_2_	PLA + 0.10% MoS_2_	PLA + 0.25% MoS_2_	PLA + 0.50% MoS_2_	PLA + 1.0% MoS_2_
Longitudinal infill pattern							
ΔRe	-	−9%	−35%	−14%	−17%	−27%	−42%
Honeycomb infill pattern							
ΔRe	-	15%	5%	−15%	−24%	16%	−3%

**Table 9:** Melt flow index (MFI) properties of PLA and PLA/MoS_2_ composites.

	Neat PLA	PLA + 0.025% MoS_2_	PLA + 0.05% MoS_2_	PLA + 0.10% MoS_2_	PLA + 0.25% MoS_2_	PLA + 0.50% MoS_2_	PLA + 1.0% MoS_2_
ΔMFI	-	30%	51%	81%	119%	219%	416%

**Table 10:** Results of thermogravimetric analysis.

	1% Mass Loss [°C]		Onset Temperature [°C]		Temperature at the Maximum Rate of Mass Loss [°C]	
Conditions	N_2_	Air	N_2_	Air	N_2_	Air
Neat PLA	304.8	307.9	343.0	348.3	361.2	367.0
PLA + 0.025% MoS_2_	247.3	255.3	336.8	338.7	358.3	360.5
PLA + 0.05% MoS_2_	257.5	256.8	336.0	337.9	358.5	359.9
PLA + 0.1% MoS_2_	252.2	213.0	336.6	332.3	356.4	355.9
PLA + 0.25% MoS_2_	272.2	274.5	335.1	329.9	355.8	354.4
PLA + 0.5% MoS_2_	256.6	185.9	339.8	333.2	359.4	355.8
PLA + 1% MoS_2_	274.4	220.3	340.2	329.9	360.4	352.2

**Table 11:** Results of differential scanning calorimetry analysis.

	T_g_ [°C]	T_cc_ [°C]	T_m_ [°C]
Neat PLA	62.0	124.5	154.5
PLA + 0.025% MoS_2_	64.9	115.1	149.7
PLA + 0.05% MoS_2_	65.6	113.0	156.1
PLA + 0.1% MoS_2_	66.9	115.0	153.4
PLA + 0.25% MoS_2_	65.7	112.5	155.1
PLA + 0.5% MoS_2_	66.1	115.1	152.7
PLA + 1% MoS_2_	65.1	112.1	155.1

**Table 12:** Contact angle analysis.

Sample	Contact Angle [°]
Neat PLA	63.7 ± 3.9
PLA + 0.025% MoS_2_	70.8 ± 2.3
PLA + 0.05% MoS_2_	71.2 ± 1.6
PLA + 0.1% MoS_2_	71.0 ± 3.0
PLA + 0.25% MoS_2_	70.9 ± 0.7
PLA + 0.5% MoS_2_	71.1 ± 0.8
PLA + 1% MoS_2_	66.9 ± 1.7

**Table 13:** HDT results.

	Heat Deflection Temperature [°C]	
	Longitudinal	Honeycomb
Neat PLA	54.50 ± 0.20	55.76 ± 0.27
PLA + 0.025% MoS_2_	58.77 ± 0.65	56.20 ± 0.17
PLA + 0.05% MoS_2_	58.00 ± 0.10	56.54 ± 0.12
PLA + 0.1% MoS_2_	57.86 ± 0.07	56.19 ± 0.09
PLA + 0.25% MoS_2_	57.87 ± 0.06	56.10 ± 0.20
PLA + 0.5% MoS_2_	57.63 ± 0.06	53.90 ± 0.10
PLA + 1.0% MoS_2_	57.47 ± 0.15	56.23 ± 0.11

**Table 14:** Results of PLA wear tests cooperating with C45 steel (v = 0.34 m/s, T_0_ = 23 °C, p = 0.11 MPa, Ra = 0.35–0.45 µm).

	Neat PLA	PLA + 0.025% MoS_2_	PLA + 0.05% MoS_2_	PLA + 0.10% MoS_2_	PLA + 0.25% MoS_2_	PLA + 0.50% MoS_2_
Δ*I_h_*	-	184%	301%	188%	198%	457%

**Table 15:** Results of friction tests in PLA pair with C45 steel (v = 0.34 m/s, T_0_ = 23 °C, p = 0.11 MPa, Ra = 0.35–0.45 µm).

	Neat PLA	PLA + 0.025% MoS_2_	PLA + 0.05% MoS_2_	PLA + 0.10% MoS_2_	PLA + 0.25% MoS_2_	PLA + 0.50% MoS_2_
Δ*µ*	-	19%	−4%	−4%	−6%	0%

## Data Availability

Not applicable.
